# Resuscitative Endovascular Balloon Occlusion of the Aorta (REBOA) for Refractory Upper Gastrointestinal Bleeding: A Case Series

**DOI:** 10.7759/cureus.92334

**Published:** 2025-09-15

**Authors:** Shun Muramatsu, Hiromu Okano, Hiroshi Okamoto

**Affiliations:** 1 Department of Critical Care Medicine, St. Luke's International Hospital, Tokyo, JPN; 2 Department of Social Medical Sciences, Graduate School of Medicine, International University of Health and Welfare, Tokyo, JPN

**Keywords:** aortic occlusion, hemorrhage, hemorrhagic shock, intensive care unit (icu), upper gastrointestinal hemorrhage

## Abstract

While resuscitative endovascular balloon occlusion of the aorta (REBOA) is a recognized life-saving intervention for hemorrhagic control in trauma, evidence supporting its use in managing refractory upper gastrointestinal bleeding (UGIB) is still limited. However, REBOA holds considerable potential for providing crucial hemodynamic stabilization in refractory UGIB cases where conventional therapies fail to achieve adequate hemostasis. We present cases of three elderly patients (88F, 72M, 74M) with refractory UGIB and profound hemorrhagic shock unresponsive to initial resuscitation. REBOA was emergently deployed at the bedside in the intensive care unit (ICU) using anatomical landmarks and ultrasound guidance, achieving rapid circulatory stabilization and enabling subsequent definitive hemostasis by surgical or endoscopic methods. No REBOA-related complications such as organ dysfunction or ischemia were observed. Our experience suggests REBOA may serve as a safe and effective bridge to hemostasis in select patients with refractory UGIB, even when deployed in an ICU setting without angiography. Further research is needed to establish standardized protocols and define optimal patient selection.

## Introduction

Hemorrhage is the leading cause of preventable death. Resuscitative endovascular balloon occlusion of the aorta (REBOA) has emerged as a minimally invasive adjunct for managing noncompressible torso hemorrhage where traditional external hemostasis is ineffective [1]. REBOA involves the insertion of a catheter via the femoral artery and temporary balloon inflation within the aorta to occlude distal blood flow, thereby maintaining perfusion to proximal vital organs such as the brain and myocardium while reducing ongoing hemorrhage. Originally developed as a life-saving intervention in trauma, REBOA has increasingly been applied in nontraumatic hemorrhagic conditions, including obstetric catastrophic bleeding, ruptured abdominal aortic aneurysms, and visceral artery aneurysms [2-4]. It is gaining recognition as a bridging strategy to stabilize circulation temporarily until definitive hemostasis - via endoscopy, endovascular embolization, or surgery - can be achieved.

While several reports have demonstrated the effectiveness of REBOA in nontraumatic intra-abdominal hemorrhage, evidence regarding its use in managing upper gastrointestinal bleeding (UGIB) that is refractory to conventional therapies (refractory UGIB) remains limited [2-4]. Most UGIB cases are managed with pharmacologic therapy and endoscopy. However, in cases of refractory UGIB with profound hemodynamic instability unresponsive to initial resuscitation, achieving rapid hemorrhage control before irreversible organ damage occurs can be extremely challenging. Definitive hemostatic procedures, such as endoscopy or surgery, may require patient stabilization or transfer, during which ongoing bleeding can be fatal. At present, there are no established criteria or standardized protocols for REBOA use in refractory UGIB, and its precise safety profile, efficacy, and specific indications in this context remain to be fully elucidated. REBOA has the potential to address the critical time gap by providing temporary hemodynamic stabilization as a bridge to definitive hemostasis.

The aim of this case series is to illustrate the potential utility and feasibility of REBOA deployed by intensivists in the intensive care unit (ICU) - even without immediate angiography suite capabilities - as a life-saving temporary measure for refractory UGIB complicated by profound hemodynamic shock. Ultimately, this case series aims to expand the clinical perspective on REBOA beyond trauma and support further investigation into its use for critical gastrointestinal hemorrhage.

## Case presentation

Case 1

**Figure 1:**
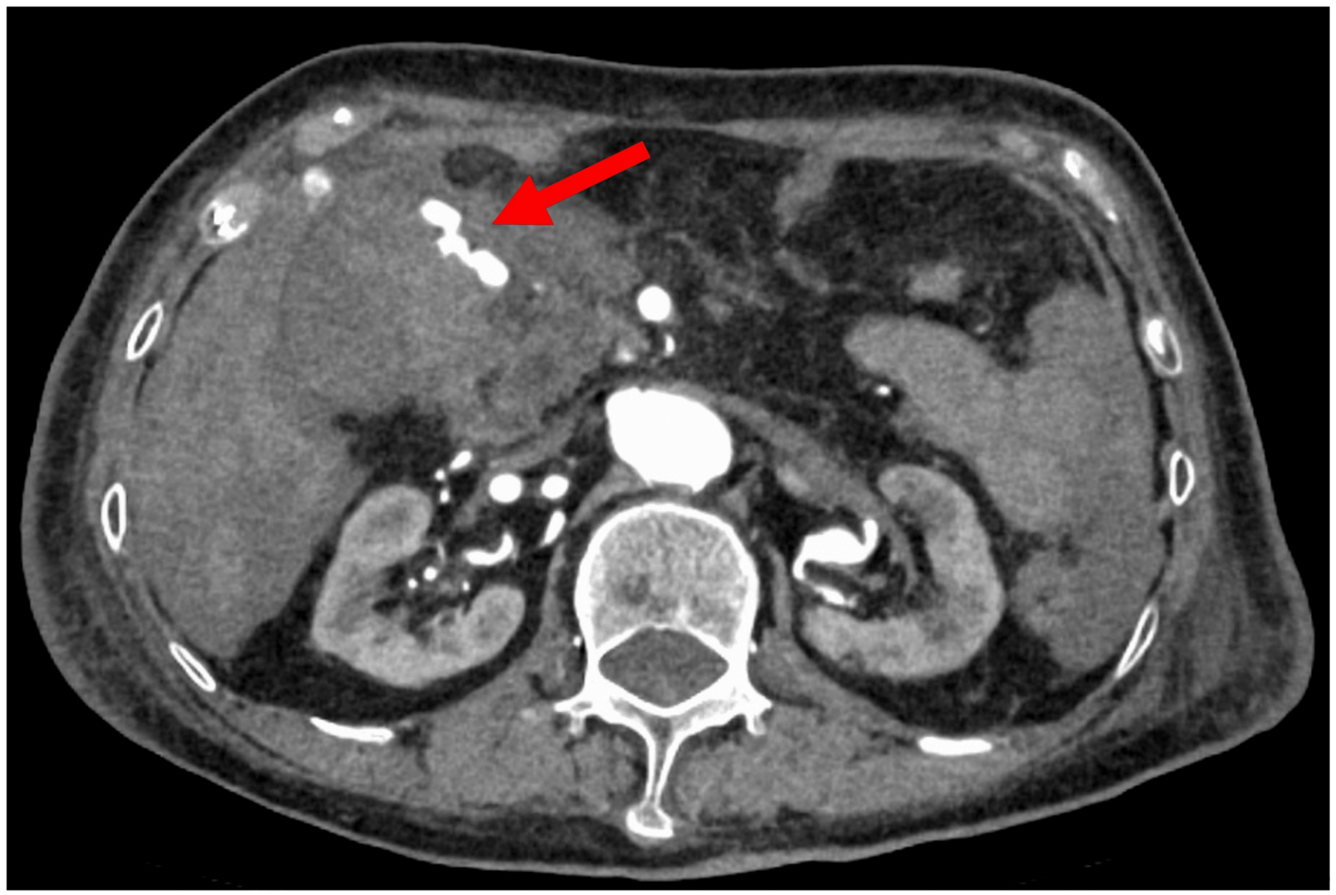
Contrast-enhanced abdominal computed tomography (Case 1) The red arrow indicates active extravasation from the gastroduodenal artery.

**Figure 2:**
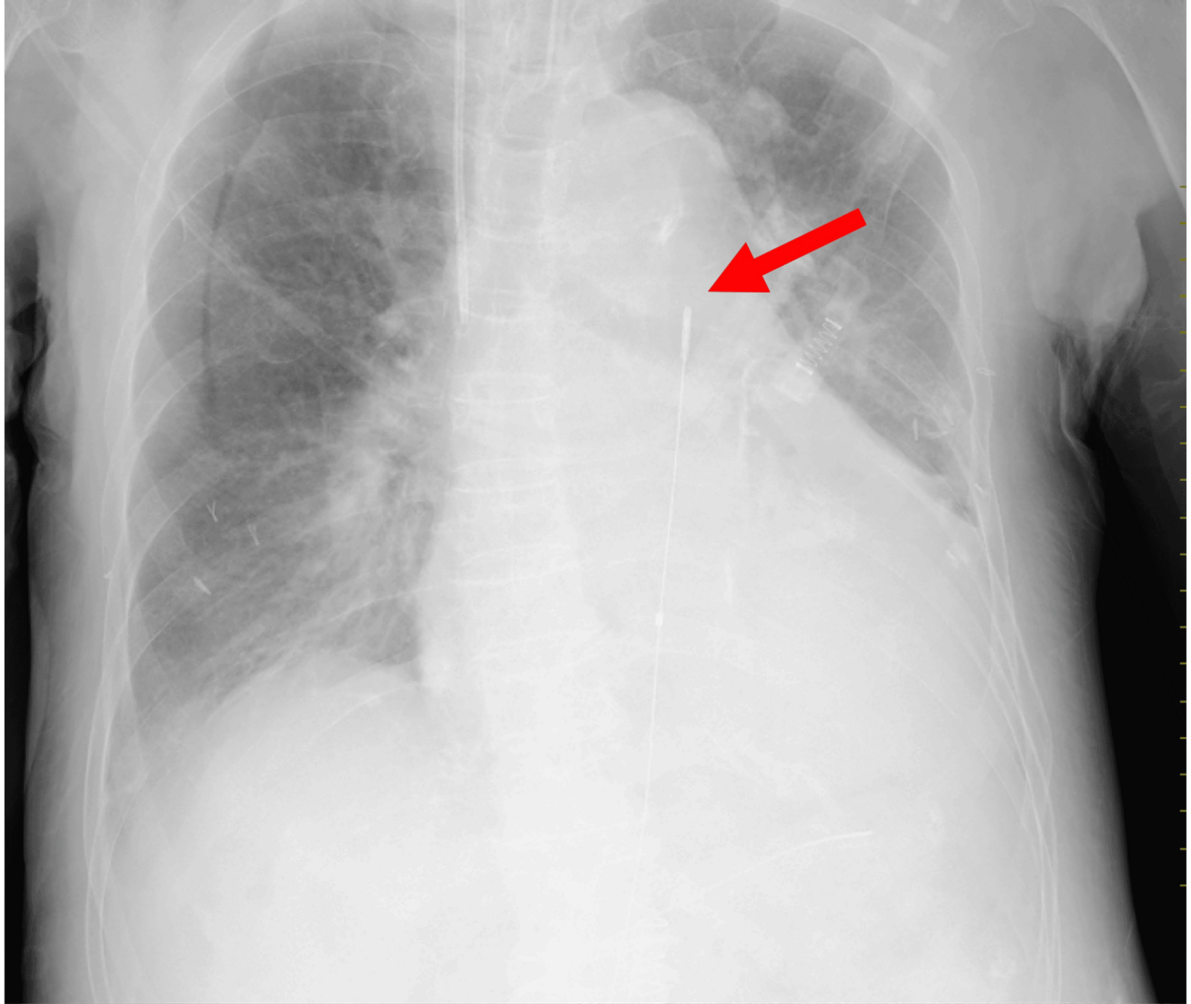
Chest X-ray demonstrating the REBOA catheter (Case 1) The red arrow indicates the tip of the REBOA catheter, which is appropriately positioned. REBOA: Resuscitative endovascular balloon occlusion of the aorta

An 88-year-old woman with no notable family history was admitted to the ICU following an uneventful laparoscopic distal gastrectomy for gastric body cancer. On postoperative day (POD) 8, she developed sudden hypotension, abdominal pain, and vomiting, suggestive of postoperative bleeding. Rapid fluid resuscitation provided temporary stabilization; contrast-enhanced abdominal computed tomography (CT) subsequently revealed active extravasation from the gastroduodenal artery (Figure 1). Despite administration of 1,000 mL of crystalloids and norepinephrine at 0.15 µg/kg/min, the patient’s blood pressure remained 60/37 mmHg (mean arterial pressure (MAP) 45 mmHg) with a heart rate of 120 bpm, indicating persistent circulatory collapse consistent with refractory hemorrhage. This prompted the emergent placement of a REBOA catheter (Rescue Balloon-ER; Tokai Medical Products, Aichi, Japan), which was inserted via the right femoral artery. Proper positioning of the catheter in Zone I of the descending aorta was confirmed by chest X-ray (Figure 2). With hemodynamic support from REBOA, endovascular embolization was attempted but failed to control the bleeding, ultimately necessitating an emergency laparotomy for definitive hemostasis. Her postoperative course was uneventful, with no REBOA-related complications such as organ dysfunction or reperfusion injury. She was discharged from the ICU on POD 16, and her condition remained stable thereafter. She was subsequently transferred to a rehabilitation facility.

Case 2

**Figure 3:**
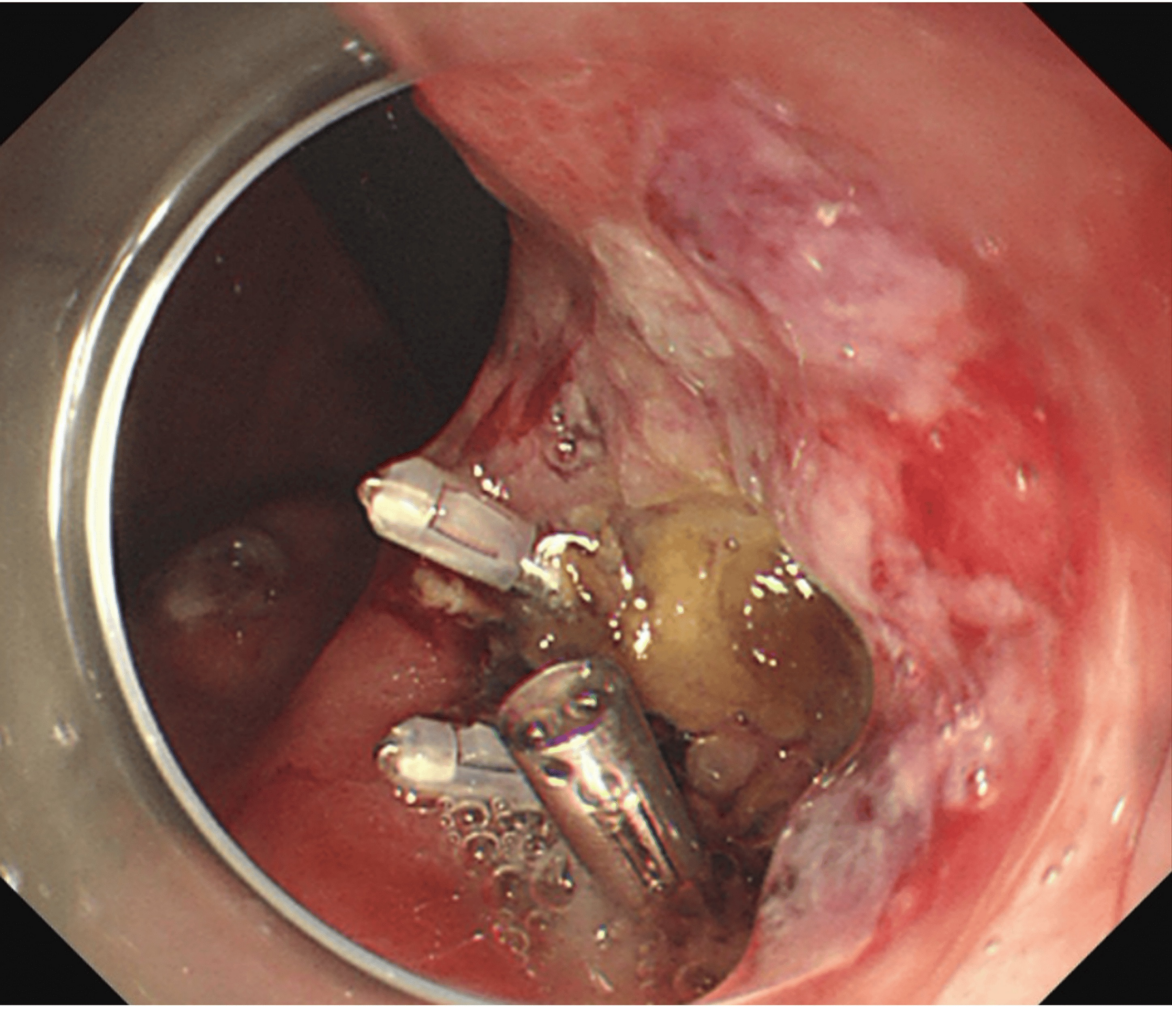
Upper gastrointestinal endoscopy (Case 2) Endoscopic clipping of an ulcer on the lesser curvature of the mid-gastric body.

**Figure 4:**
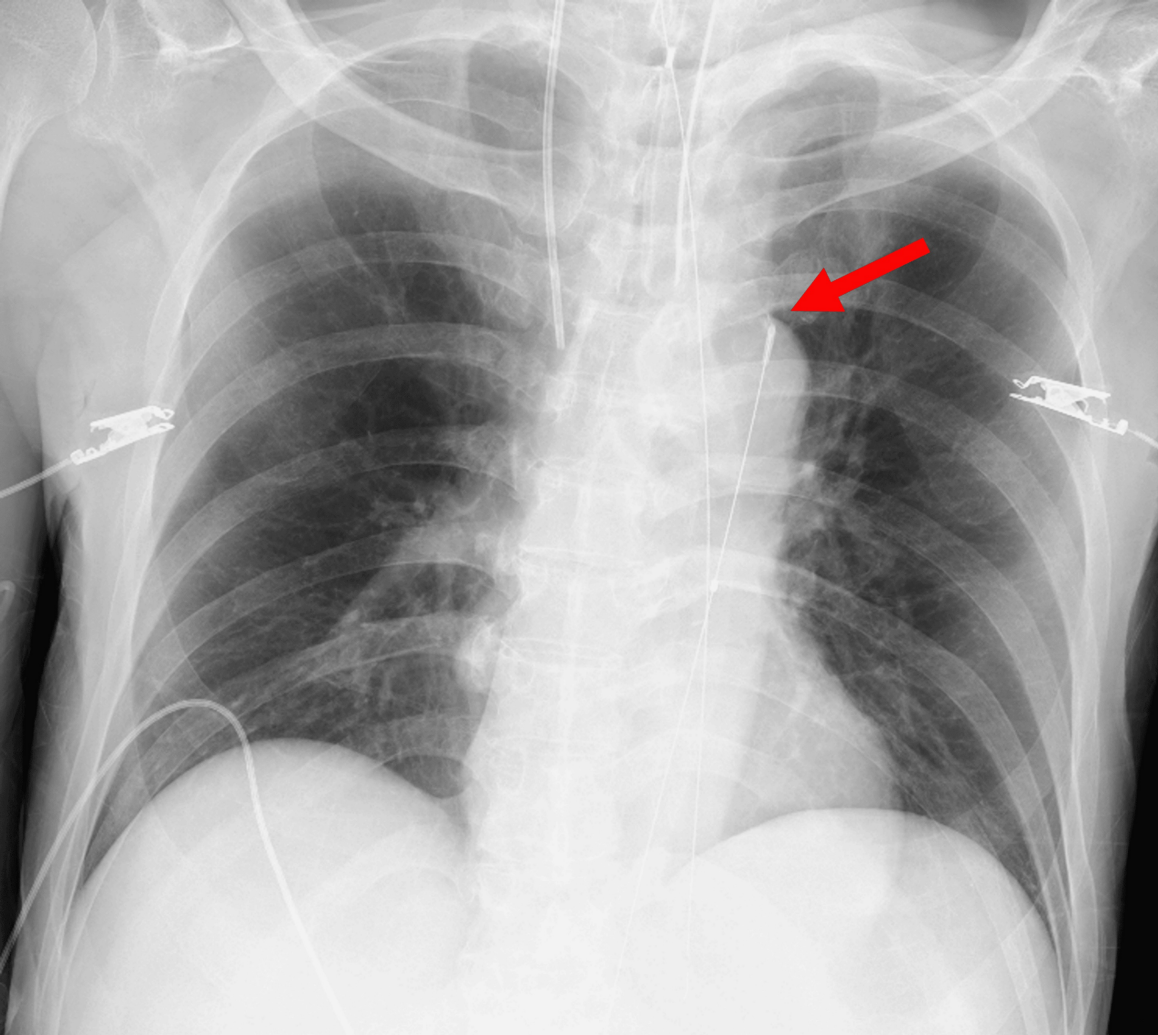
Chest X-ray demonstrating the REBOA catheter (Case 2) The red arrow indicates the tip of the REBOA catheter, which is appropriately positioned. REBOA: Resuscitative endovascular balloon occlusion of the aorta

A 72-year-old man with no notable family history presented with epigastric pain and melena. Initial upper gastrointestinal endoscopy revealed an ulcer on the lesser curvature of the mid-gastric body, and endoscopic clipping was performed for hemostasis (Figure 3). He was subsequently admitted to the ICU. On the following day, second-look endoscopy showed rebleeding from the same site, accompanied by hypotension. Despite administration of 1,000 mL of crystalloids and norepinephrine at 0.3 µg/kg/min, the patient’s blood pressure remained 62/37 mmHg (MAP 45 mmHg) with a heart rate of 140 bpm, indicating ongoing circulatory collapse consistent with refractory hemorrhage. Because urgent endoscopic reintervention was deemed at that moment, a REBOA catheter (Rescue Balloon-ER; Tokai Medical Products, Aichi, Japan) was emergently inserted via the right common femoral artery as a bridge to definitive hemostasis. The REBOA catheter, positioned in Zone I, rapidly achieved circulatory stabilization (Figure 4), facilitating subsequent endovascular embolization, which successfully achieved hemostasis. No REBOA-related complications such as organ dysfunction or reperfusion injury were observed. The patient was discharged from the ICU on day 5 of admission, and he was discharged home. He continues to be followed up in our outpatient clinic.

Case 3

**Figure 5:**
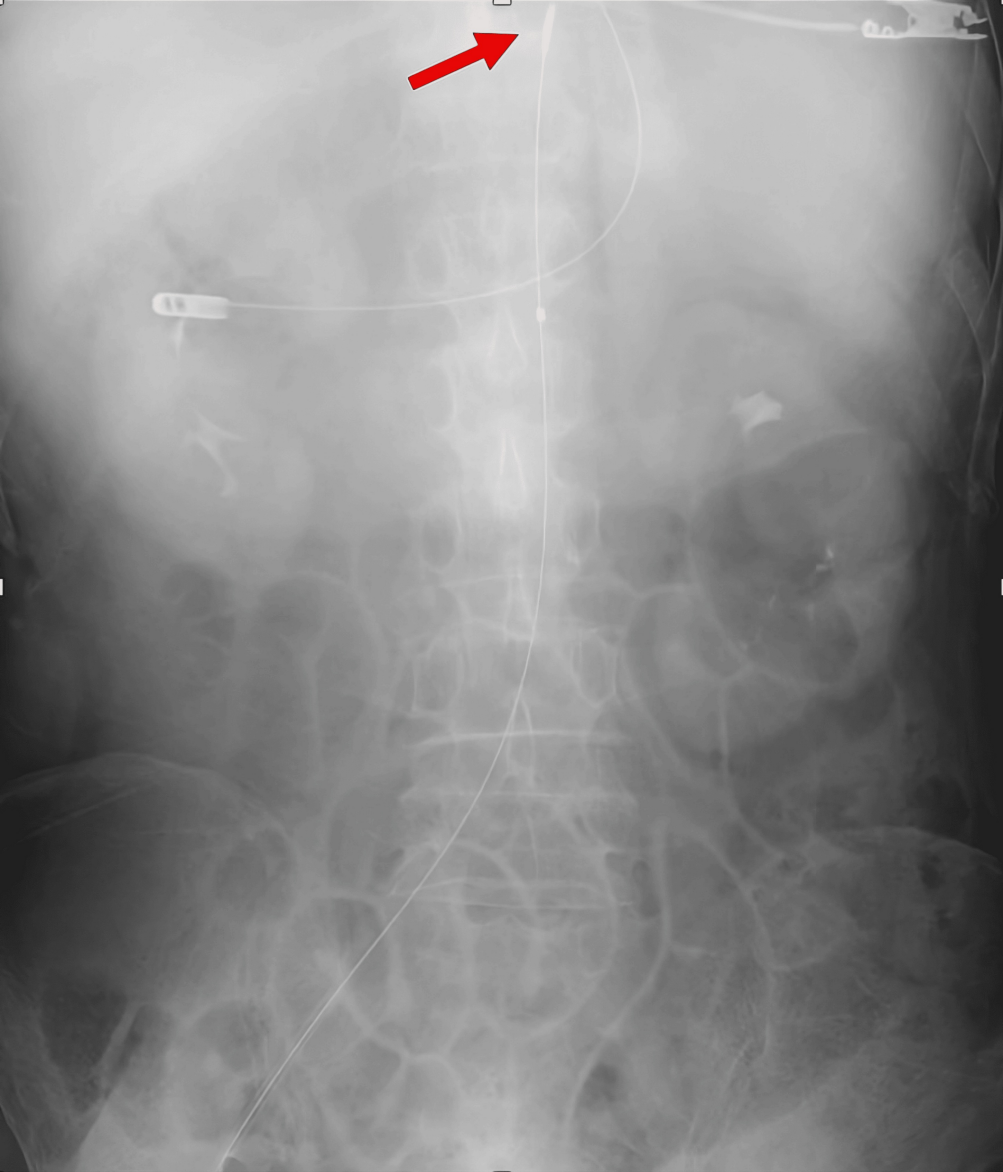
Chest X-ray demonstrating the REBOA catheter (Case 3) The red arrow indicates the tip of the REBOA catheter, which is appropriately positioned. REBOA: Resuscitative endovascular balloon occlusion of the aorta

**Figure 6:**
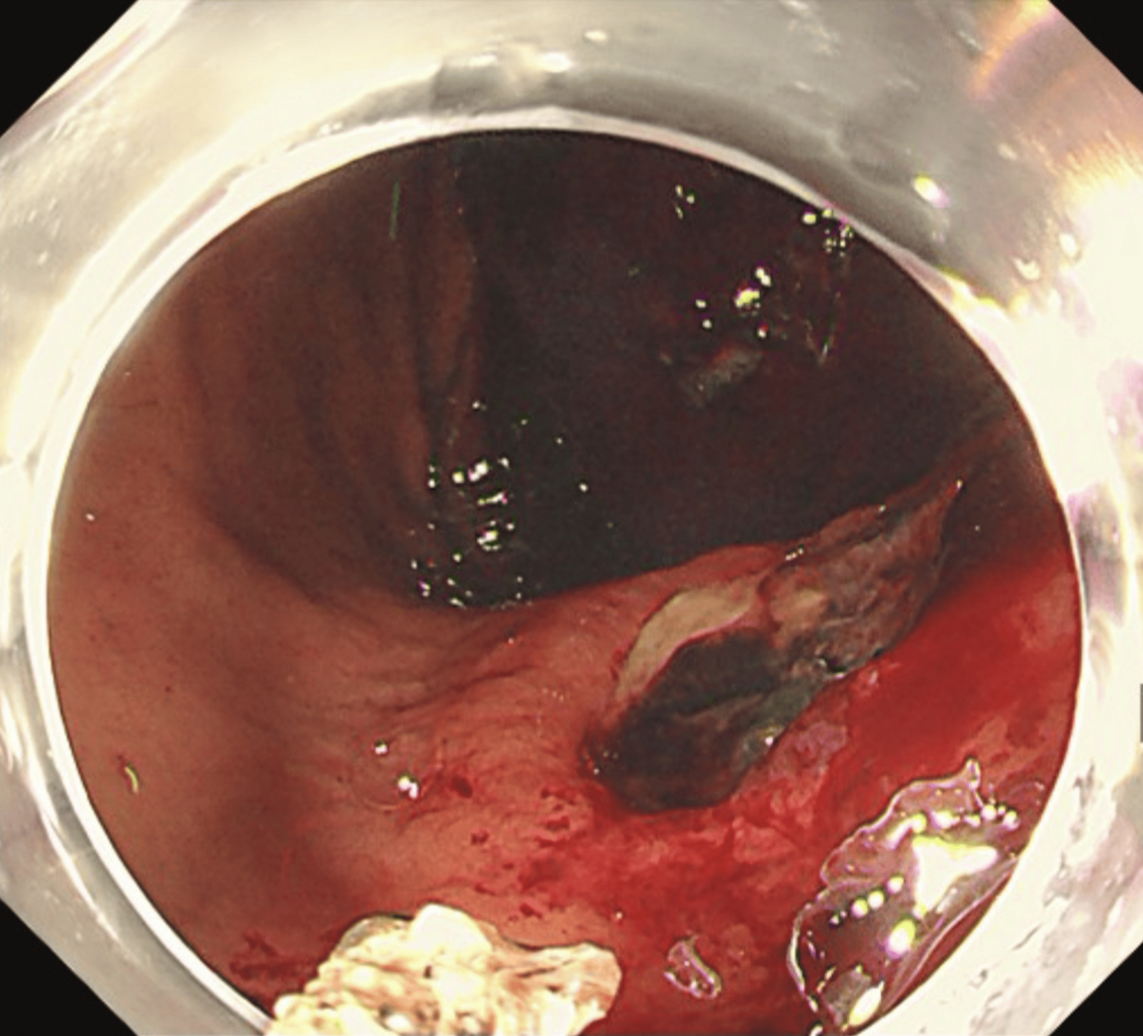
Upper gastrointestinal endoscopy (Case 3) An ulcer on the lesser curvature of the lower gastric body without active bleeding.

A 74-year-old man with no notable family history was emergently transported to the hospital with hematemesis and was subsequently admitted to the ICU. On hospital day 3, he experienced massive hematemesis and developed hypotension without an identifiable trigger. Despite aggressive resuscitation - including a massive transfusion protocol (total RBCs 8 units, fresh frozen plasma (FFP) 8 units, platelets 20 units by that time), crystalloid boluses, and norepinephrine at 0.45 µg/kg/min - his blood pressure remained 46/32 mmHg (MAP 37 mmHg) with a heart rate of 138 bpm. This persistent instability prompted emergent insertion of a REBOA catheter (Rescue Balloon-ER; Tokai Medical Products, Aichi, Japan) via the right common femoral artery, positioned in Zone I (Figure 5). Following circulatory stabilization achieved by REBOA, upper gastrointestinal endoscopy was performed, revealing an ulcer on the lesser curvature of the lower gastric body without active bleeding at that time (Figure 6). Conservative management was therefore initiated.

His condition remained stable until hospital day 16, when he developed sudden hypotension (49/23 mmHg; MAP 32 mmHg) with large-volume bleeding from the nasogastric tube. At that time, he was receiving norepinephrine at 0.4 µg/kg/min with ongoing blood transfusion and albumin therapy. Because immediate endoscopy was not feasible during nighttime hours at our institution, REBOA was reinserted via the right common femoral artery and positioned in Zone I as a bridge to definitive hemostasis. This once again achieved rapid circulatory stabilization, facilitating a repeat endoscopy. This second endoscopy identified active bleeding from the previously noted ulcer, and endoscopic clipping was successfully performed to achieve definitive hemostasis. No REBOA-related complications, including organ dysfunction or reperfusion injury, were observed. The patient was discharged from the ICU on hospital day 18, and his condition remained stable thereafter. He was subsequently transferred to a rehabilitation facility.

Summary of the cases

Table 1 shows the clinical characteristics, REBOA procedural details, and hemostatic interventions for all three cases.

**Table 1:** Summary of the three cases In all three cases presented, REBOA was placed in Zone I without fluoroscopic guidance. In each case, REBOA placement led to hemodynamic stabilization, allowing for safe performance of hemostatic interventions. No REBOA-related organ dysfunction or reperfusion injury was observed in any of the patients. REBOA: Resuscitative endovascular balloon occlusion of the aorta

	Case 1	Case 2	Case 3
Pre-REBOA lowest blood pressure (mmHg)	60/37	62/37	46/32
Pre-REBOA heart rate (bpm)	120	140	138
REBOA insertion zone	Zone I	Zone I	Zone I
Aortic occlusion time (minutes)	30	40	30
Guidance for insertion	Ultrasound-guided	Ultrasound-guided	Ultrasound-guided
REBOA-related complication	None	None	None
Source of bleeding	Gastroduodenal artery	Lesser curvature ulcer, mid-gastric body	Lesser curvature ulcer, lower gastric body
Definitive hemostatic intervention	Emergency laparotomy for hemostasis	Endovascular embolization	Endoscopic clipping

## Discussion

In this case series, we present three patients with refractory UGIB who developed profound hemorrhagic shock unresponsive to the initial resuscitation and were subsequently managed with REBOA in the ICU. In all patients, temporary hemodynamic stabilization was achieved, enabling a safe transition to definitive hemostatic intervention. Although REBOA has traditionally been used for traumatic hemorrhage, its indications have recently expanded to include nontraumatic causes such as obstetric hemorrhage, ruptured abdominal aortic aneurysms, and visceral artery aneurysms [1,2,3,5]. These cases further highlight the potential utility of REBOA as a life-saving bridging intervention in appropriately selected patients with refractory UGIB.

REBOA use for nontraumatic hemorrhagic shock, including gastrointestinal bleeding, has increased in recent years; however, its use remains infrequent in clinical practice [6]. Several reports have described REBOA use for nonvariceal UGIB, including those by Sano et al. and Matsumura et al., with all reporting favorable hemodynamic responses [5,7]. However, in most of these reports, REBOA was performed either in emergency departments or during interhospital transport, rather than during active resuscitation in the ICU [5]. Our case series offers a unique perspective by demonstrating the feasibility of REBOA deployment at the bedside during resuscitation in the ICU. This approach may particularly benefit patients with refractory UGIB who remain hemodynamically unstable despite intensive care. Compared with earlier reports, our patients received earlier intervention in a controlled ICU environment, under direct monitoring.

Our patients experienced no complications related to REBOA, such as intestinal ischemia, acute kidney injury, or limb ischemia. This contrasts with previous studies in trauma patients, which have reported complication rates of 5-20% [7], often linked to prolonged occlusion times, poor vascular access, or incorrect balloon placement. In our series, three factors likely contributed to the absence of complications: short occlusion times (30-40 minutes), rapid transition to definitive therapy, and the operator’s skill in ultrasound-guided vascular access and balloon positioning. These findings support the idea that minimizing occlusion time and ensuring technical accuracy can help reduce these risks. Regarding our technique, we used ultrasound to access the femoral artery and positioned the balloon using anatomical landmarks, without fluoroscopic guidance. Zone I occlusion, proximal to the celiac artery, was deemed necessary to control hemorrhage originating from the upper gastrointestinal tract. Although this fluoroscopy-free method raises concerns about safety, several studies have shown that experienced teams can perform REBOA safely in critical care settings using the same approach [8,9]. Our results support these findings, as all procedures succeeded without complications. However, this technique requires excellent knowledge of vascular anatomy and ultrasound, particularly in unstable patients.

This case series demonstrates the potential feasibility and safety of REBOA for refractory UGIB in select patients. It is important to emphasize that urgent endoscopy remains the first-line diagnostic and therapeutic intervention for UGIB. In the present cases, REBOA served solely as a temporary bridging strategy to stabilize circulation when endoscopy or interventional radiology could not be performed immediately due to severe hemodynamic instability or resource constraints, after which patients proceeded to definitive hemostasis. However, its expanded application critically requires further standardization and research. REBOA remains an invasive and resource-intensive procedure that necessitates careful patient selection. To broaden its use for nontraumatic bleeding, especially UGIB, standardized protocols, structured training, and simulation practice will be essential to ensure safe and consistent outcomes. Despite the limitation of this being a three-case series, our findings suggest that bedside REBOA is a feasible and safe intervention for refractory UGIB in the ICU. Reports on assessments of multiple patients are necessary to validate these results and establish best practices for nontraumatic indications.

## Conclusions

This case series provides compelling preliminary evidence that REBOA, when implemented by trained intensivists in an ICU setting, can serve as a safe and effective adjunctive intervention for refractory UGIB. REBOA may provide temporary hemodynamic stabilization and facilitate definitive hemostasis in critically ill patients with refractory UGIB. Further studies should be conducted to establish standardized protocols and clarify appropriate patient selection criteria.
